# Supplementary material for: Most bothersome symptom in migraine and probable migraine: A population-based study
Source: PLoS One. 2023 Nov 29;18(11):e0289729. doi: 10.1371/journal.pone.0289729 (PMC10686452; doi:10.1371/journal.pone.0289729)
Supplement: S1 Table — (DOCX) [file pone.0289729.s001.docx]

**S1 Table. Sociodemographic distribution of the total Korean population, survey participants, and cases of migraine and PM**

|  | | Survey participants,  n (%) | Total population,  n (%) | *p*-value | Migraine,  n, % (95 % CI) | PM  n, % (95 % CI) | *p*-value |
| --- | --- | --- | --- | --- | --- | --- | --- |
| **Sex** | | | | | | | |
|  | Men | 1,551 (51.2) | 15,529,105 (51.2) | >0.999 | 41, 2.6 (1.8–3.4) | 130, 8.4 (7.0–9.8) | <0.001 |
|  | Women | 1,479 (48.8) | 14,778,651 (48.8) |  | 129, 8.7 (7.2–10.2) | 209, 14.1 (12.6–15.9) |  |
| **Age (years)** | | | | | | | |
|  | 20–29 | 673 (22.2) | 6,719,119 (22.1) | >0.999 | 34, 5.1 (3.4–6.7) | 69, 10.3 (8.0–12.6) | 0.280 |
|  | 30–39 | 685 (22.6) | 6,839,377 (22.6) |  | 44, 6.4 (4.6–8.3) | 86, 12.6 (10.1–15.0) |  |
|  | 40–49 | 819 (27.0) | 8,208,901 (27.1) |  | 60, 7.3 (5.5–9.1) | 99, 12.1 (9.9–14.3) |  |
|  | 50–59 | 853 (28.2) | 8,540,359 (28.2) |  | 32, 3.8 (2.5–5.0) | 85, 10.0 (8.0–12.0) |  |
| **Size of residential area** | | | | | | | |
|  | Large city | 1,364 (45.0) | 13,667,248 (45.1) | 0.488 | 65, 4.8 (3.6–5.9) | 162, 11.9 (10.2-13.6) | 0.546 |
|  | Medium-to-small city | 1,376 (45.4) | 12,143,800 (40.1) |  | 86, 6.3 (5.0–7.5) | 147, 10.7 (9.1–12.3) |  |
|  | Rural area | 290 (9.6) | 4,496,708 (14.8) |  | 19, 6.6 (3.7–9.4) | 30, 10.3 (6.8–13.9) |  |
| **Education level** | | | | | | | |
|  | High school or lower | 1,212 (40.0) | 12,395,872 (40.9) | 0.897 | 66, 5.4 (4.1–6.7) | 133, 11.0 (9.2–12.7) | 0.760 |
|  | College or higher | 1,818 (60.0) | 17,911,884 (59.1) |  | 104, 5.7 (4.7–6.8) | 206, 11.3 (9.8–12.8) |  |
| **Total** | | 3,030 (100.0) | 30,307,756 (100.0) |  | 170, 5.6 (4.8–6.4) | 339, 11.2 (10.1–12.3) |  |

Note: Data are expressed as n (%) or median (25 %–75 %)

PM, probable migraine; CI, confidence interval
