# Supplementary material for: Most bothersome symptom in migraine and probable migraine: A population-based study
Source: PLoS One. 2023 Nov 29;18(11):e0289729. doi: 10.1371/journal.pone.0289729 (PMC10686452; doi:10.1371/journal.pone.0289729)
Supplement: S2 Table — (DOCX) [file pone.0289729.s002.docx]

**S2 Table. Distribution of associated symptoms in participants with migraine and those with PM**

|  | | Migraine, n=170 | PM, n=339 |
| --- | --- | --- | --- |
| **Reported symptoms of the participants, n (%)** | | | |
|  | Nausea | 120 (70.6) | 282 (83.2) |
|  | Vomiting | 82 (48.2) | 132 (39.0) |
|  | Photophobia | 125 (73.5) | 146 (43.1) |
|  | Phonophobia | 137 (80.6) | 166 (49.0) |
| **Number of associated symptoms, n (%)** | | | |
|  | 1 | 9 (5.3) | 96 (28.3) |
|  | 2 | 80 (47.1) | 135 (39.8) |
|  | 3 | 29 (17.1) | 64 (18.9) |
|  | 4 | 52 (30.6) | 42 (12.4) |

PM, probable migraine
