# Supplementary material for: Most bothersome symptom in migraine and probable migraine: A population-based study
Source: PLoS One. 2023 Nov 29;18(11):e0289729. doi: 10.1371/journal.pone.0289729 (PMC10686452; doi:10.1371/journal.pone.0289729)
Supplement: S3 Table — (DOCX) [file pone.0289729.s003.docx]

**S3 Table. Use of acute medication classes in the migraine and PM groups**

|  | Combination analgesics, n (%) | NSAIDs, n (%) | Simple analgesics, n (%) | Triptans, n (%) | Tramadol, n (%) | Unknown, n (%) | All acute treatments, n (%) |
| --- | --- | --- | --- | --- | --- | --- | --- |
| Migraine, n=170 | 60 (35.3) | 27 (15.9) | 78 (45.9) | 1 (0.6) | 2 (1.2) | 3 (1.8) | 127 (74.7) |
| PM, n=339 | 107 (31.6) | 44 (13.0) | 133 (39.2) | 1 (0.3) | 4 (1.2) | 0 (0.0) | 213 (62.8) |

NSAIDs: nonsteroidal anti-inflammatory drugs; PM, probable migraine
